# Annotations

**Published:** 1887-07-16

**Authors:** 


### Annotations.

The Hospitals Association and its future work.

The Council of The Hospitals Association met last week for general business, and to arrange a programme for the coming session, which will commence in October. Several schemes of great practical moment occupied the attention of the Council, and will form the subjects of papers by competent authorities. Among these, not the least important are The Nurses' Registration scheme, The National Pension Fund, The Amelioration of the Condition of Midwives, and the general question of Hospital Architecture and construction. There is a strong tendency on the part of learned and scientific societies to be purely or largely speculative, and to carefully avoid those questions which demand work and results. By its very constitution The Hospitals Association is precluded from sinking into a mere talking society. Where so much and such important work is needed to be done, an Association which merely talked would make itself ridiculous. There are many capable persons, both men and women, among the members of the Society, and they should consider it an urgent duty to attend all its meetings and give to its discussions an intelligent, vigorous, and practically useful character. Any reader, or indeed anyone who takes a practical interest in hospitals and their welfare, should write to the Secretary at Norfolk House, W.C., and ask him to send a copy of the Annual Report and Constitution. Suggestions of subjects for discussion or offers of papers to be read during the winter session, should also be sent in without delay, as the Council are now considering the programme for the evening meetings.

Cook your Milk.

Some years ago an ingenious young physiologist attached to one of the Scotch Universities, discovered a number of minute creatures in samples of sugar, and thereupon the cry of "death in the sugar-bowl" was raised. Since that time the active researches of similar young men have made us feel uncomfortable about water, vegetables, pork, milk, and a variety of other substances. We have been accustomed to regard milk as one of the safest, most nutritious, and easily digestible of foods. A recent outbreak of scarlet fever quickened curiosity and effort in the direction of the milk supply, and now a generalisation seems to have been arrived at on sufficient grounds that on the whole the best course to take with milk as an article of food is to make an invariable rule of cooking it. According to the judgment of some very competent and other less distinguished authorities, we ought no more to take uncooked milk than uncooked kidney or raw chicken and ham. Of course the world has been digesting raw milk for six thousand years at any rate, and the fluid still holds its own in general estimation. That being so, we need not be very greatly alarmed about the recent scares and the supposed new discoveries. The generalisations even of men of undoubted scientific authority are sometimes hasty, and are often modified or superseded by further discoveries. Still it is not a difficult or very troublesome thing to boil milk, and, by way of being on the safe side, it will be better for a time to make a rule of doing so.

Who Nurses the Sick Nurse?

Nurses are but human, and will fall sick at times. Who takes care of them? Does anybody? No doubt most of them can go home to their friends for a brief period in order to recruit, but that entails cost to the friends and loss to the nurse. The occupation of sick nursing demands qualities of a high order, as intelligence, steady devotion to duty, and strict probity. Women possessed of these attributes, who give the very best years of their life to their most trying duties, should be as free from all anxiety as they can possibly be made. Not only should they have a modest pension on which to retire when they can work no more, but adequate provision should be made against temporary breakdowns, which are sure to occur in a certain proportion of cases. The profession of nursing should be worked on the lines of the public services in some respects, and will be so worked if nurses can be made to understand the need and value of co-operation. A breakdown to one person is a very serious thing, but if the burden of it be borne by ten thousand shoulders instead of one, it becomes pecuniarily a mere trifle. Matrons especially, who have both more education and experience than nurses, should consider it an imperative duty not only to push forward the Pension Fund scheme, but also to promote co-operation among women of their own order for mutual help and defence against the inevitable casualties of life.

Bald Heads.

"One touch of nature" makes bald people all over the world sympathise with one another Dr. Jackson, of New York, delivers a lecture on baldness, and forthwith *The Standard* and other English papers seize upon what they are sure will be interesting "copy," and publish the cream of the discourse. How many persons of fifty will be soothed by Dr. Jackson's opinion that baldness is not necessarily a sign of age! It appears that it is the men who get bald, and of these the more intellectual and cultured portion. Great scholars and thinkers are often bald; the coming man is to be quite bald. The hairy scalp is declared to be a relic of pristine animality, which will gradually be shuffled off as development advances. We would wager a hundred pounds to a pinch of snuff that Dr. Jackson himself was bald at twenty. We roundly confess our disbelief in all this rubbish. Too much brain work may tend to baldness, but it tends to many other evils as well. We cannot afford to lose our animal vigour. In our judgment a sound and robust body is the necessary accompaniment of a strong and well-balanced mind.

